# Corrigendum: Cascading failures in coupled networks with both inner-dependency and inter-dependency links

**DOI:** 10.1038/srep34431

**Published:** 2016-10-10

**Authors:** Run-Ran Liu, Ming Li, Chun-Xiao Jia, Bing-Hong Wang

Scientific Reports 6: Article number: 25294; 10.1038/srep25294 published online: 05 04 2016; updated: 10 10 2016.

This Article contains typographical errors.

In the Results section under subheading ‘Scale-free networks’,

“We keep *R*^*B*^ constant in eq. (4), and check the behaviours of the order parameter *R*^*A*^”

should read:

“We keep *R*^*B*^ constant in eq. (3), and check the behaviours of the order parameter *R*^*A*^”

In the same section,

“Similar to the coupled random networks, 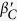 and 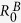 can be solved numerically by letting 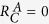 in eq. (4),”

should read:

“Similar to the coupled random networks, 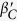 and 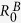 can be solved numerically by letting 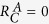 in eq. (3),”

In the same section,

“At the other tricritical point *β*_*c*_, the conditions of continuous and discontinuous percolation transitions are satisfied simultaneously, i.e., *β*_*c*_ makes the first and the second order derivative of eq. (4) with respective *S*^*A*^ hold at the percolation transition point *p*_*c*_”.

should read:

“At the other tricritical point *β*_*c*_, the conditions of continuous and discontinuous percolation transitions are satisfied simultaneously, i.e., *β*_*c*_ makes the first and the second order derivative of eq. (3) with respective *R*^*A*^ hold at the percolation transition point *p*_*c*_”.

In the same section,

“Similar to random networks, we cannot get the analytical expressions for the first order percolation transition points, but they can be solved numerically by eq. (4)”.

should read:

“Similar to random networks, we cannot get the analytical expressions for the first order percolation transition points, but they can be solved numerically by eqs. (3) and (4)”.

